# Inhibitory Effects of *Rabdosia rubescens* in Esophageal Squamous Cell Carcinoma: Network Pharmacology and Experimental Validation

**DOI:** 10.1155/2022/2696347

**Published:** 2022-11-10

**Authors:** Ruoyang Lin, Xianfan Lin, Jinming Wu, Tanzhou Chen, Zhiming Huang

**Affiliations:** Department of Gastroenterology and Hepatology, The First Affiliated Hospital of Wenzhou Medical University, Wenzhou 325000, China

## Abstract

Esophageal squamous cell carcinoma (ESCC) is one of the most frequently occurring diseases in the world. *Rabdosia rubescens* (RR) has been demonstrated to be effective against ESCC; however, the mechanism is unknown. The primary gene modules related to the clinical characteristics of ESCC were initially investigated in this research using weighted gene co-expression network analysis (WCGNA) and differential expression gene (DEG) analysis. We employed network pharmacology to study the hub genes linked with RR therapy on ESCC. A molecular docking simulation was achieved to identify the binding activity of central genes to RR compounds. Lastly, a chain of experimentations was used to verify the inhibitory effect of RR water extract on the ESCC cell line *in vitro*. The outcomes revealed that CCNA2, TOP2A, AURKA, CCNB2, CDK2, CHEK1, and other potential central targets were therapeutic targets for RR treatment of ESCC. In addition, these targets are over-represented in several cancer-related pathways, including the cell cycle signaling pathway and the p53 signaling pathway. The predicted targets displayed good bonding activity with the RR bioactive chemical according to a molecular docking simulation. *In vitro* experiments revealed that RR water extracts could inhibit ESCC cells, induce cell cycle arrest, inhibit cell proliferation, increase P53 expression, and decrease CCNA2, TOP2A, AURKA, CCNB2, CDK2, and CHEK1. In conclusion, our study reveals the molecular mechanism of RR therapy for ESCC, providing great potential for identifying effective compounds and biomarkers for ESCC therapy.

## 1. Introduction

Esophageal cancer is one of the most common types of malignancy in the world. It is the 8^th^ frequent cause of death in adults and 6^th^ common cause of death in youngsters. China has one of the highest incidence and fatality rates of esophageal cancer globally [1, 2]. The majority of esophageal carcinomas are squamous cell carcinomas (ESCCs). Despite tremendous breakthroughs in the treatment of esophageal cancer, there is currently no therapeutic option, and the 5-year survival rate remains poor [3]. Given this, further research on the treatment of esophageal squamous cell carcinoma is still needed. TCM (traditional Chinese medicine) has been used in Asia for over two thousand years [4]. TCM has attracted more and more attention due to its minor side effects and practical anti-cancer effect.

*Rabdosia rubescens* (Hemsl.) Hara (Lamiaceae), abbreviated as RR, is a traditional Chinese medicine used to cure stomach aches, pharyngitis, sore throat, cough, and trauma. Because of its anti-tumor properties, this herb is gaining popularity [5]. RR contains various active ingredients, including diterpenoids, flavonoids, phenolic compounds, triterpenoids, and volatile oils [6]. RR has an anti-tumor influence on various types of tumors, such as breast cancer [7, 8], liver cancer [9], gastric cancer [10], and colon cancer [11]. Current reports have confirmed that RR has an important inhibitory effect on esophageal squamous cell carcinoma [12–14].

**Figure 1:**
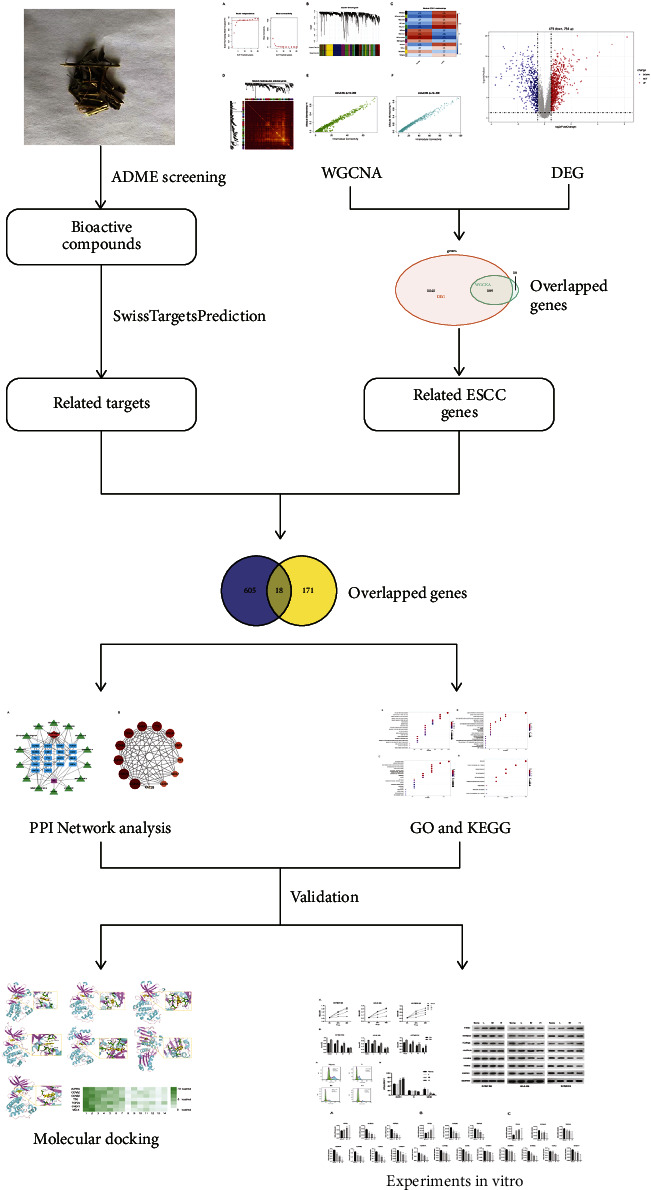
The technical strategy for this article.

Network pharmacology is an emerging field based on systems biology theory, carries out a network analysis of biological systems, and chooses particular signal nodes to project multi-target drug molecules. For complex systemic diseases like cancer, where single-target interventions are ineffective, web-based pharmacological approaches are beneficial because they differ from traditional therapies exploring multi-target therapies for such conditions [15]. Network pharmacology focuses on modulating many signaling pathways, which corresponds to traditional Chinese medicine's multi-component and multi-target properties [16, 17]. Through network pharmacology, this paper intends to investigate the putative molecular mechanism of *Rabdosia rubescens*'s anti-esophageal squamous cell cancer activity and to build the theoretical groundwork for future clinical applications of *Rabdosia rubescens*.  Figure 1 represents the flowchart for this study.

## 2. Materials and Methods

### 2.1. Collection Gene Targets of ESCC

GSE161533 from the GEO database (https://www.ncbi.nlm.nih.gov/gds) was used in this study, a total of 56 cases samples, including 28 normal and 28 ESCC samples. First, we did WGCNA. WGCNA is a technique for assessing gene expression patterns across several samples. It can classify genes based on their expression patterns and investigate the relationship between modules and certain features or phenotypes. It examines relationships between modules and specific qualities or phenotypes, screens models that are substantially connected with traits, and examines genes in modules to identify target genes for research. In this study, the R package “WGCNA” (version 3.6.1 for Windows) was used to test whether the genes in the samples needed to be filtered. When the output results were “TRUE,” the next step was to build a co-expression network. The ESCC and normal samples were taken as the trait data of WGCNA to look for the hub gene associated with ESCC. Second, to screen out genes associated with ESCC, we used the limma package for differential analysis between normal samples and ESCC patients. The differential filter condition is |Log2 FC| > 0.5, *p* value< 0.05. Third, the intersection of the most relevant module gene after the first step and differential tumor gene after the second step was taken to draw a Venn diagram. Finally, all of the targets mentioned above were consistent with gene names and UniProt IDs using the “*Homo sapiens*” filter in the UniprotKB (https://www.uniprot.org/) database.

### 2.2. RR Bioactive Compound Collection

All the RR components were retrieved from BATMAN (https://bionet.ncpsb.org/batman-tcm/) database [18], TCMID (https://119.3.41.228:8000/tcmid) database, TCM (https://tcm.cmu.edu.tw/) Database@Taiwan [19], and the Encyclopedia of Traditional Chinese Medicine (ETCM, https://www.nrc.ac.cn:9090/ETCM/index.php) [20].

The Spatial Data Format (SDF) format files or the simplified molecular-inputline-entry specification (SMILES) information of the active substances were obtained from PubChem (https://pubchem.ncbi.nlm.nih.gov) and ChemSpider (https://www.chemspider.com). The compounds were chosen based on their qualities utilizing established screening parameters such as gastrointestinal absorption and drug-likeness standards. All of the chemicals, as mentioned earlier, were entered into the SwissADME database (https://www.swissadme.ch/) for absorption, distribution, metabolism, and excretion (ADME) testing.

### 2.3. RR Drug Target Prediction

Bioactive ingredients obtained by screening were predicted through Swiss Target Prediction (https://www.swisstargetprediction.ch/), and the target with prediction score greater than 0 was taken as the drug target, and the condition was set as “*Homo sapiens.*”

The gene name and gene ID were confirmed by UniProt (https://www.uniprot.org/).

### 2.4. Construction of Networks and Analysis

Venn analysis was used to determine overlap between RR and ESCC target genes. For protein-protein interaction (PPI) network development, the intersection genes were punched into the STRING database (11.0 version). For high confidence basis, “*Homo sapiens*” was chosen for protein interactions, and a score of >0.4 was selected as the low confidence basis. Cytoscape 3.7.2 software was then used to visualize and analyze the data, and hub genes were identified based on their degree value, which was calculated by the connectivity among nodes. A drug-component-target-disease network map was created and viewed using the Cytoscape 3.7.2 platform.

### 2.5. Pathway Enrichment Analysis Using Gene Ontology (GO) and Kyoto Encyclopedia of Genes and Genomes (KEGG)

The GO and KEGG analyses were conducted using Bioconductor package in R software (version 3.6.1 for Windows) package (*Q* value < 0.05, *p* value < 0.05), and the results were displayed in bubble charts.

### 2.6. Molecular Docking Analysis

To model molecular docking, the bioactive components of RR were utilized as ligands and the target proteins were utilized as receptors. Ligand files were acquired via PubChem. Molecular modeling was performed using Schrodinger software. The ligand structures were hydrogenated, structure optimized, and energy minimized in Schrodinger software. The crystal structures of CCNA2, TOP2A, AURKA, CCNB2, CDK2, CHEK1, and TTK target proteins were acquired from the RCSB protein data library. On the Maestro 11.9 platform, the protein structures were processed. The protein was processed utilizing Schrodinger's Protein Preparation Wizard to remove the crystal water, add the missing hydrogen atom, repair the lost bond information, and fix the missing peptide. Finally, the energy of the protein was reduced, and its geometric structure was improved. In the Schrodinger Maestro software, the Glide module processes and optimizes virtual filtering. The receptors were pretreated, optimized, and optimized again (constraint minimization using OPLS3e force field). All of the compounds were synthesized using the LigPrep module's default parameters. The produced receptors were loaded into the Glide module during testing to identify the correct location in the receptor grid generation. The protein's original ligand was chosen as the center of mass of the 12 Å box. The compound-target protein interaction model was examined to determine the interactions with each target residue, such as hydrogen bonding, *π*-*π* interaction, and hydrophobic interaction. The docking scores of the compounds were compared to predict whether the compounds to be screened have specific active effects.

### 2.7. Experimental Validation

#### 2.7.1. Extraction Methods of Components and Cell Culture

The extraction method of RR was according to the previous literature [21]. Water extracts were diluted to low (1 g/L), medium (5 g/L), and high concentrations (10 g/L). KYSE150 esophageal squamous cell carcinoma cells were obtained from Cobier Biotechnology Co. LTD (Nanjing, China). ECA109 and KYSE510 esophageal squamous cell carcinoma cells were purchased from the Cell Bank of the Chinese Academy of Sciences (Shanghai, China). KYSE150 cell line was cultured in Dulbecco's modified eagle medium (DMEM, HyClone, Logan, UT, USA) and ECA109 and KYSE510 cell lines were cultured in Roswell Park Memorial Institute‐1640 (RPMI‐1640, HyClone, Logan, UT, USA) with 10% fetal serum and 1% penicillin streptomycin combination (Solarbio, Beijing, China) at 37°C in an incubator with 5% CO_2_ and 95% O_2_.

#### 2.7.2. Cell Counting Kit-8 (CCK8) Assay

The growing cells were digested by trypsin (Solarbio, Beijing, China) and counted under the microscope to make a cell suspension of 1∼5 × 10^4^ cells/ml. 96 well culture plates were taken, and each cell plate was inoculated with 3 same holes as multiple holes, 1–5 × 10^3^ cells/well. Then, all cells were distributed into 4 groups: the standard group (None group), the low-dose water extract group (WL group), the medium-dose water extract group (WM group), and the high-dose water extract group (WH group). The standard group cells were cultured in a 100 *μ*l medium as blank control; the other groups were treated with the corresponding concentration of water extract (1 g/L, 5 g/L, and 10 g/L). After overnight culture at 37°C, each well was treated with a 20 *μ*l CCK8 solution from Beyotime (Shanghai, China). After that, the wells were incubated for a further 4 hours at 37°C with 5% CO_2_. The supernatant was gently cultivated in the suction hole. For suspended cells, the supernatant should be sucked after centrifugation. The absorbance of each group at 490 nm was measured with a microplate reader (Perlong, Beijing, China).

#### 2.7.3. Soft Agar Colony Formation Assay

The preheated 1.2% soft agar was mixed with culture medium in the 6-well cell culture plates, waiting for solidification at room temperature. KYSE150 cells in each group at logarithmic growth stage were blown into single cells and suspended in 10% fetal bovine serum medium for later use. The cell suspension in each group blended with the mixture of the preheated 0.7% soft agar, and culture medium was inoculated into the well at the gradient density of cells in each well and gently rotated to make the cells evenly dispersed. The cells were incubated at 37°C with 5% CO_2_ and saturated humidity for 2–3 weeks. It was often observed that culture was terminated when visible clones appeared. We took photos and calculated the number of clones.

#### 2.7.4. Analysis of the Cell Cycle

All cells were carefully collected and preserved in anhydrous ethanol for 24 h at 4°C. After rinsing the suspended cells with cold PBS, they were treated for 30 minutes at room temperature with 1 mg/mL RNase. Cells were then incubated in the dark for 10 minutes with a 400 *μ*L propidium iodide (PI) solution at a concentration of 50 *μ*g/mL. Flow cytometry (BD Accuri C6) was used to identify the data, and then they were analyzed using FlowJo (Version 10).

#### 2.7.5. Quantitative RT-PCR

The total RNA of all cells was extracted using the TRIzol reagent according to the manufacturer's procedure (Invitrogen, Carlsbad, CA, USA). Reverse transcriptase M-MLV (Fermentas, CA) and TransStart Tip Green qPCR SuperMix Kit (Thermo Fisher Scientific, USA) were used for reverse transcription and quantitative PCR. The sequences of primers were as follows: Cellular tumor antigen p53 (TP53) (F: 5′-CATTTGCACCTACCTCAC-3', R: 5′-TTGACAACTCCCTCTACC-3′), Cyclin A2 (CCNA2) (F: 5′-ATGAGACCCTGCATTTGG-3′, R: 5′- ACTGTTGGAGCAGCTAAG-3′),DNA-topoisomerase2-alpha (TOP2A) (F: 5′-TGACTTTACAACCCAAGAG-3′, R: 5′-TTCACCCAGTTTAGTATGC-3′), Aurora kinase A (AURKA) (F: 5′-GGGACCTCATTTCAAGAC-3′, R: 5′-CTGGCTCAAGGATTTCTC-3′), G2/mitotic-specificcyclin-B2 (CCNB2) (F: 5′-TGACTATTAGGCGAACTG -3′, R: 5′-GGTTGAACTGGAACTTTG-3′),Cyclin-dependent kinase 2 (CDK2) (F: 5'-AACAAGTTGACGGGAGAG-3′, R: 5′-AAGAGGAATGCCAGTGAG-3′), Serine/threonine-protein kinase Chk1 (CHEK1) (F: 5′ AGGAGTATTCTGACTGGAAAG 3′, R: 5′ GCTGATGGATTCTCAACTAAG 3′), and Glyceraldehyde-3-phosphate dehydrogenase (GAPDH) (F: 5′ CACCCACTCCTCCACCTTTG 3′, R: 5′ CCACCACCCTGTTGCTGTAG 3′).

#### 2.7.6. Western Blot Analysis

RIPA (Solarbio, Beijing, China), containing protease and phosphatase inhibitors, extracted protein from all cells. The BCA kit (Thermo Fisher Scientific, USA) was used to determine the protein content. SDS-PAGE was used to separate the protein samples, which were then transferred to polyvinylidene difluoride membranes. The membranes were blocked for 1 hour at 37°C with 5% skimmed milk or 5% bovine serum albumin in 0.1% Tween 20 in 1× Tris-buffered saline (TBS/T). The membranes were incubated overnight at 4°C with the primary antibodies TP53, CCNA2, TOP2A, AURKA, CCNB2, CDK2, and CHEK1 (Bioss, Beijing, China). After incubation with the main antibody, the blots were washed away 3 times in TBS-T and incubated for two hours at room temperature with a horseradish-conjugated goat anti-rabbit or anti-mouse antibody (Beyotime, Shanghai, China). The film was exposed to the gel imaging system after the ECL exposure solution was dropped upon it. A C± hemiDocTM XRS + Imaging System was used to examine the grey value of each antibody strip (Bio-Rad, Shanghai, China).

#### 2.7.7. Data Analysis

All experiments were conducted in triplicate technical replicates, and the data were presented as mean ± SD. To determine statistical significance, a one-way ANOVA with SPSS 25.0 was utilized. The statistical data chart was created using the GraphPad Prism software (Version 8.0, USA). *p* < 0.05 was considered as statistically significant, while the highly significant difference level was set at *p* < 0.01.

## 3. Result

### 3.1. Identification of ESCC Genes

**Figure 2:**
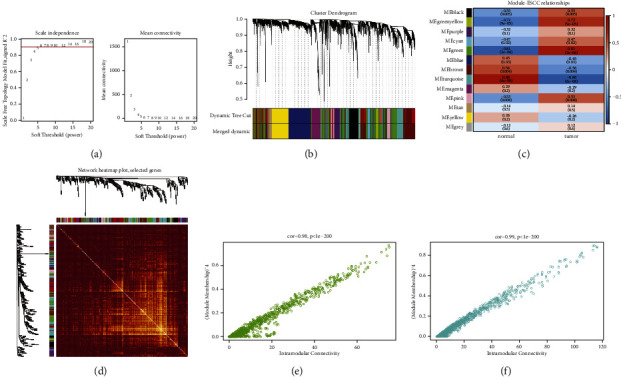
WGCNA analysis for ESCC genes. (a) Scale-free soft threshold distribution diagram. The horizontal axis represents the power value of the weight parameter, the vertical axis in the left figure represents the square of the correlation coefficient between log(k) and log(p(k)) in the corresponding network, and the vertical axis in the right figure represents the mean value of all gene adjacency functions in the corresponding gene module. (b) Clustering dendrogram. (c) Heat map of correlation between modules and clinical traits. The ordinate is different module, and the abscissa is different trait. Each square represents the correlation coefficient and significance *p* value of a certain module and a certain trait. (d) Network topological overlap measure heatmap plot. The upper part of the figure is the selected gene cluster tree, the lower part is the heat map, and the horizontal and vertical colors represent different modules. (e) Green module MM and GS scatter diagrams. (f) Turquoise module MM and GS scatter diagrams.

**Figure 3:**
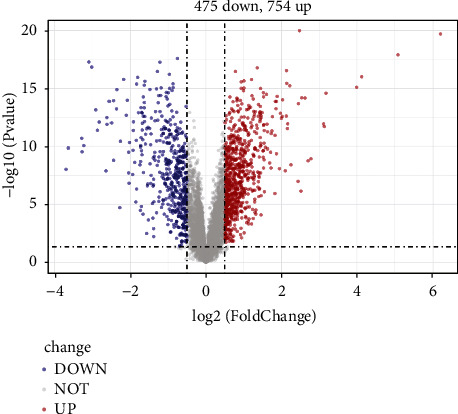
DEG analysis of GEO161533. The abscissa represents the multiple of difference, and the ordinate represents −log 10 (*p* value). The red dots are upregulated genes and the blue dots are downregulated genes.

**Figure 4:**
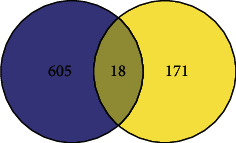
Venn diagram after WGCNA and DEG analysis.

First of all, the samples were clustered. Then, the soft threshold was determined for the data. As shown in Figure 2(a), the soft threshold was equal to 5, and R^2 was close to the threshold of 0.9 (red line). Meanwhile, mean connectivity in the right figure was also close to 0. Therefore, the optimal soft threshold was selected as 5. Then, the adjacencies between genes were calculated, and the similarity between genes was calculated according to the adjacencies, and the dissimilarity coefficient between genes was deduced, and the cluster tree between genes was obtained accordingly. The minimum number of genes in each gene module was then adjusted to 50 according to the hybrid dynamic tree-cutting algorithm standard. In addition to the grey module, a total of 28 modules were gathered. MEDissThres was set to 0.2 to merge similar modules analyzed by the dynamic clipping tree algorithm. After merging, there were 13 modules in total, and the module diagram in Figure 2(b) was generated. Finally, the MEgreen module and Meturquoise revealed a high positive and negative correlation with tumor samples, with correlation coefficients larger than 0.81, both of which were significant (*p* < 0.05). These two modules are selected for subsequent analysis. To select ESCC-related vital modules, we screened the highest correlation with ESCC module (|cor| > 0.8 and *p* < 0.05), and they were Green and Turquoise modules. There were 985 genes in the Green module and 1234 genes in the Turquoise module. We made a heat map of modules and clinical traits and randomly selected 400 genes to draw gene cluster tree and heat map (Figures 2(c) and 2(d)). We carried out module membership (MM) and gene significance (GS) correlation calculations and made a scatter diagram, as shown in Figures 2(e) and 2(f). The critical module genes were screened according to the standard of |MM| > 0.8 and |GS| > 0.8. After screening, 97 genes were screened by the Green module and 115 genes by the Turquoise module. Differential analysis using the limma package showed a total of 1229 significantly differentially expressed genes between standard samples and ESCC patients, including 754 upregulated genes and 475 downregulated genes. A volcano map was made, as shown in Figure 3. To identify genes associated with ESCC disease, we identified 207 key module genes (the key module shared five genes) and 1229 differential genes. The intersection of the two gene sets was chosen to generate 189 genes, and the Venn diagram displayed in Figure 4 was created.

### 3.2. ESCC Genes Targeted by RR

**Figure 5:**
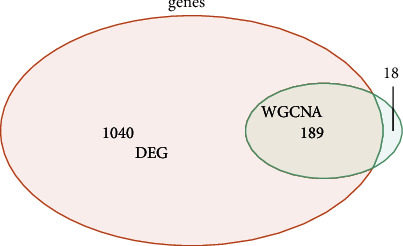
Venn diagram of related targets of RR and ESCC.

The practical components of RR were searched in the BATMAN database, TCMID database, TCM Database@Taiwan, and ETCM database for screening and imported into the SwissADME database for ADME screening. Swiss Target Prediction was used to import screened component architectures and delete components that were unable to correctly identify targets. As a result of this study, we were able to identify 21 possible active components and 623 possible pharmacological targets. There were 189 disease targets and 623 drug targets in the Venny 2.1 online software mapping tool platform, and the intersection of the two yielded 18 frequent drug-disease targets, as indicated in Figure 5. Finally, 14 active components of RR against ESCC were determined by PubChem database (7 active components that had no intersection with disease targets were deleted) (Table 1).

### 3.3. Construction and Analysis of Drug-Component-Target-Disease Network

**Figure 6:**
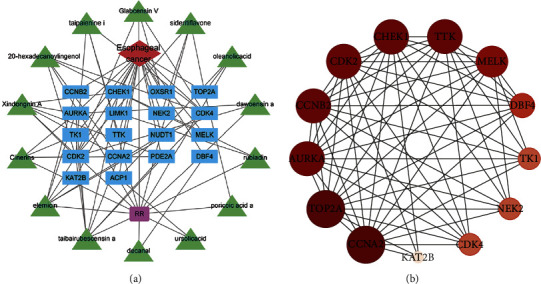
Drug-component-target-disease network and PPI network construction. (a) Drug-component-target-disease network. The red diamond represents ESCC, the green triangle represents bioactive components, the blue rectangle represents genes, and the purple rectangle represents *Rabdosia rubescens.* (b) PPI network. The node's size, color, and shade variations correspond to the degree value's size.

Fourteen potential active components and 18 common drug-disease targets of RR were input into Cytoscape software, and a “drug-components-target-disease” interaction network map was drawn, as shown in Figure 6(a) on targets, and red represents diseases. The network diagram's topology was analyzed using a network analyzer. The degree value indicated how many relationships existed between the component and the objective. The degree value indicates the component's criticality. Additionally, Table 1 shows the degree values.

### 3.4. PPI Network Construction and Tore Target Analysis

Top 18 common targets were retrieved using the STRING database. The protein type was set to “*Homo sapiens*,” and the threshold for interaction was set at 0.4. The network relationships between the target interactions were obtained and imported into the Cytoscape software to construct the protein interaction network diagram (Figure 6(b)). The node's size, color, and shade variations correspond to the degree value's size. CCNA2 (degree = 12), TOP2A (degree = 12), AURKA (degree = 12), CCNB2 (degree = 11), CDK2 (degree = 11), CHEK1 (degree = 11), TTK (degree = 11), and MELK (degree = 10) were eight high-degree targets related with numerous compounds. These network-level protein targets may be responsible 'or RR's critical therapeutic effects on ESCC.

### 3.5. Analysis of GO and Pathway Enrichment

**Figure 7:**
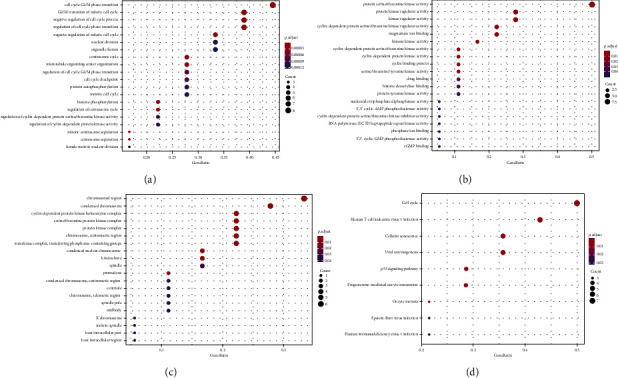
Bubble chart of analysis of GO and pathway enrichment. (a) BP results. (b) MF results. (c) CC results. (d) KEGG function enrichment.

The analysis of functional enrichment of important target genes (GO and KEGG) was performed using Bioconductor software (*Q* < value 0.05, *p* < value 0.05), and the findings were produced as a bubble chart (Figure 7).

According to the biological process (BP) data (Figure 7(a)), these targets were implicated in cell cycle G2/M phase transition, mitotic cell cycle G2/M phase transition, negative cell cycle process regulation, and cell cycle phase transition, among others. The molecular function (MF) (Figure 7(b)) included serine/threonine-protein kinase activity, protein kinase regulator activity, regular kinase activity, cyclin-dependent serine/threonine-protein kinase regulator activity, magnesium ion binding, and histone kinase activity. According to the cell composition (CC) results (Figure 7(c)), these targets were primarily involved in the chromosomal region, condensed chromosome, cyclin-dependent protein kinase holoenzyme complex, serine/threonine-protein kinase complex, protein kinase complex, chromosome, centromeric region, transferase complex, and transferring phosphorus-containing groups. KEGG pathway analysis of implicated targets was performed to elucidate the underlying processes by which RR acts on ESCC. After running the 18 genes through the R language, nine KEGG pathways were produced, generating a bubble map (Figure 7(d)). Cell cycle, human T-cell leukemia virus type 1 infection, cellular senescence, viral carcinogenesis, p53 signaling pathway, progesterone-mediated oocyte maturation, oocyte meiosis, Epstein–Barr virus infection, and human immunodeficiency virus type-1 infection were the nine KEGG pathways.

### 3.6. Molecular Docking Analysis

**Figure 8:**
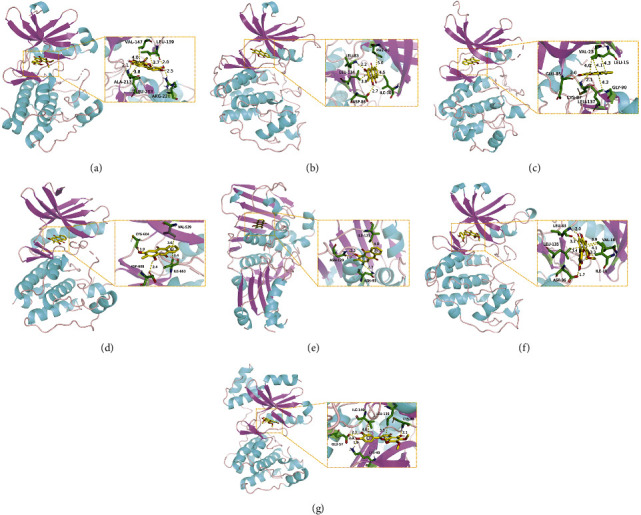
Molecular docking results of key targets and bioactive molecules. (a) AURKA-sideritiflavone. (b) CCNA2-rubiadin. (c) CCNB2-sideritiflavone. (d) TTK-rubiadin. (e) TOP2A-rubiadin. (f) CHEK1-rubiadin. (g) MELK-sideritiflavone.

**Figure 9:**
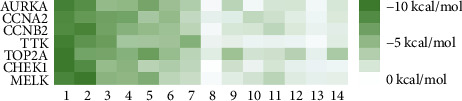
Heat map of binding capacity between key targets and the bioactive compounds.

We conducted molecular docking between 14 active components of *Rabdosia rubescens* (20-hexadecanoylingenol, cinerins, dawoensin, decanal, elemicin, glabcensin, oleanolic acid, poricoic acid A, rubiadin, sideritiflavone, taibairubescensin, taipaienine, ursolic acid, and xindongnin) and 7 target proteins (AURKA, CCNA2, CCNB2, TTK, TOP2A, CHEK1, and MELK). According to the molecular docking data, the 5 chemicals rubiadin, sideritiflavone, 20-hexadecanoylingenol, cinerins, and elemicin performed well in docking score and effective contact with protein formation. The better the binding, the lower the binding energy of the chemical to the target. The binding energies of some compounds and the five target proteins are less than −5 kcal/mol, showing solid binding effects. PyMOL 2.1 software was used to view the complex produced by the docking compound and protein (for each target, the compound with the highest score was chosen for mapping) and determine the compound and protein's binding pattern. The binding pattern reveals the amino acid residues bound by the chemical and protein pocket.  Table 2 and Figures 8 and 9 illustrate the results of the molecular docking analysis.

### 3.7. RR Reduces the Proliferation of ESCC Cells

**Figure 10:**
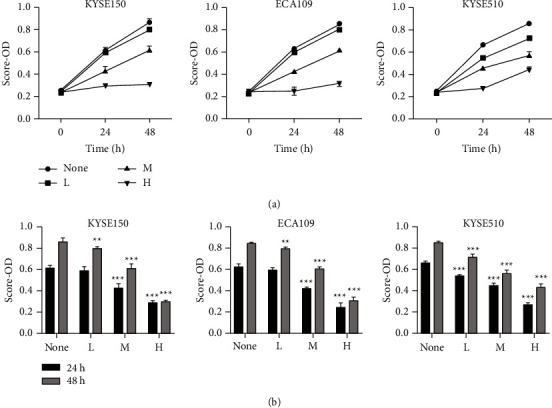
RR reduced the proliferation of ESCC cells by CCK8 assay. (a) Time and dose-dependent effects of extracts of RR treatment on the viability of ESCC cells. (b) Bar charts of OD values of each group at 24 h and 48 h (*n* = 3, mean ± SD; ^*∗*^*p* < 0.05, ^*∗∗*^*p* < 0.01, and ^*∗∗∗*^*p* < 0.001 vs. the nontreated group).

**Figure 11:**
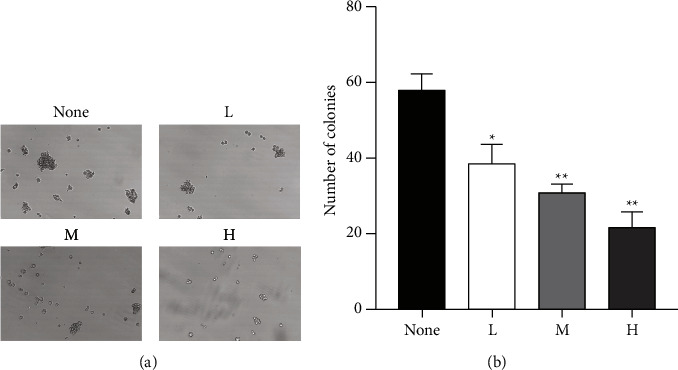
RR reduced the proliferation of ESCC cells by soft agar clone formation assay. (a) Representative image of each group. (b) Bar charts of clone cell number of each group (*n* = 3, mean ± SD; ^*∗*^*p* < 0.05, ^*∗∗*^*p* < 0.01, and ^*∗∗∗*^*p* < 0.001 vs. the nontreated group).

CCK8 assay and soft agar clone formation assay were used to detect the effect of drugs on the survival rate of ESCC cells. The CCK8 assay results showed that water extracts of RR inhibited the proliferation of ESCC cells. At 24 and 48 hours, the water extraction group's medium and high-dose groups may be able to inhibit ESCC cell proliferation. Meanwhile, the high-dose group had significantly more inhibition of proliferation than the low and medium-dose groups. The soft agar clone formation assay results also showed that low, medium, and high-dose water extraction groups of RR could all inhibit the proliferation of ESCC cells. The high-dose group had significantly more inhibition of proliferation than the low and medium-dose groups. These results demonstrated that RR reduced the proliferation of ESCC cells (Figures 10 and 11).

### 3.8. RR Inhibits the Proliferation of ESCC Cells in the G0/G1 Phase

**Figure 12:**
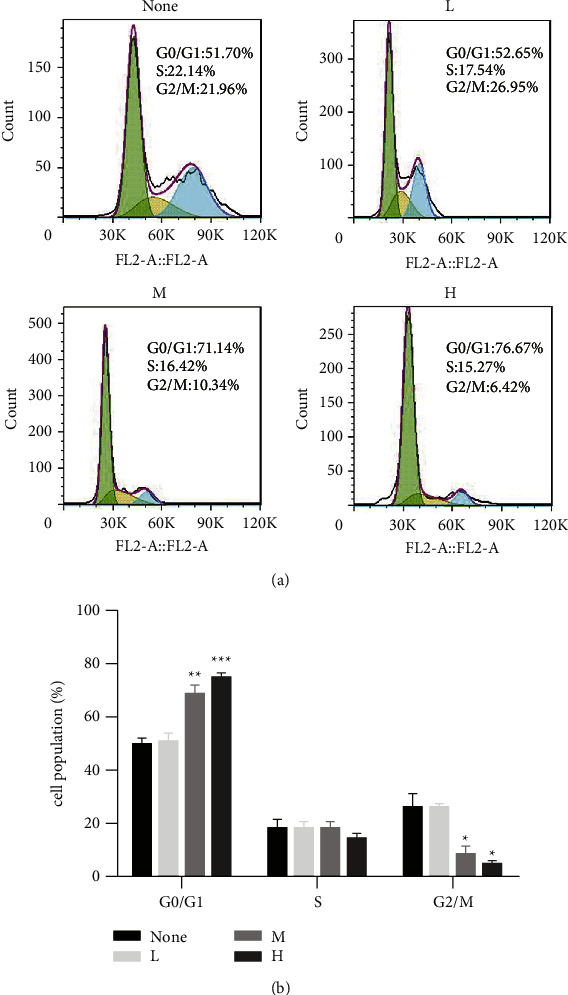
RR inhibits the proliferation of KYSE150 cells in the G0/G1 phase. (a) Cell cycle was assayed by flow cytometry. (b) Quantitative results of (a) (^*∗*^*p* < 0.05, ^*∗∗*^*p* < 0.01, and ^*∗∗∗*^*p* < 0.001 vs. the nontreated group).

To explore the effect of RR on the cell cycle of cancer cells, we used PI labelling and flow cytometry to identify the cell cycle of KYSE150 cells. Our study discovered that RR's water extracts raised the proportion of cells in the G0/G1 phase and lowered the proportion of cells in the G2/M phase, meaning that RR repressed cancer cells in the G0/G1 phase (Figure 12).

### 3.9. RR Regulates the Expression of Multiple Target Genes

**Figure 13:**
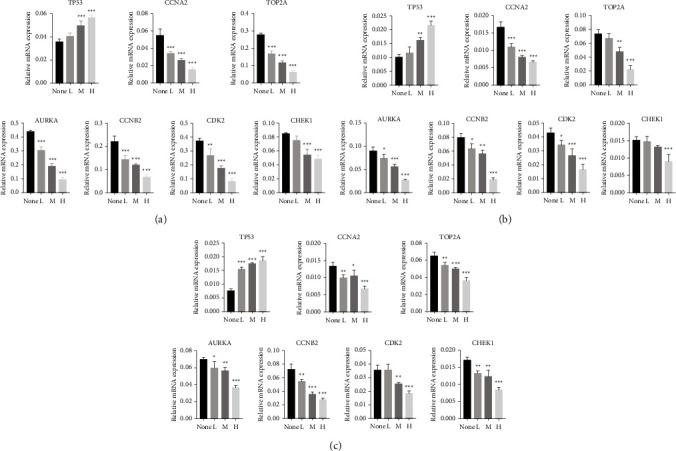
RR regulates the expression of key target genes at mRNA level. (a–c) The key target genes at mRNA level were detected by qRT-PCR. (a) KYSE150. (b) ECA109. (c) KYSE510 (^*∗*^*p* < 0.05, ^*∗∗*^*p* < 0.01, and ^*∗∗∗*^*p* < 0.001 vs. the nontreated group).

**Figure 14:**
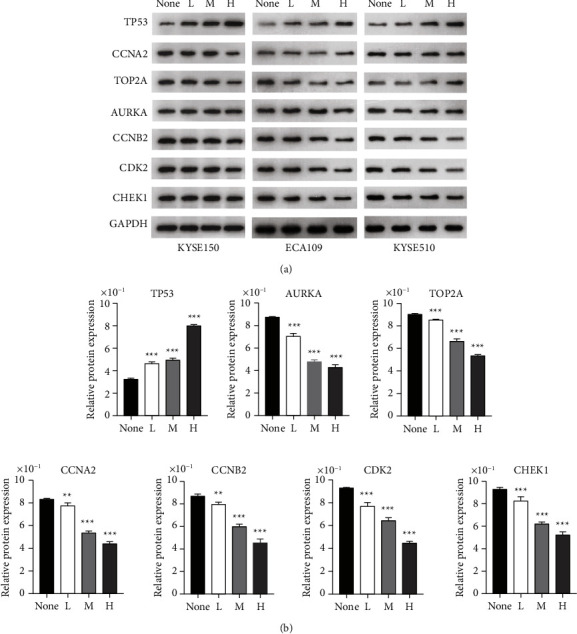
RR regulates the expression of key target genes at protein level. (a) Western blot analysis of key targets in three cell lines. (b) The quantitative analysis of gene expression in KYSE150 (^*∗*^*p* < 0.05, ^*∗∗*^*p* < 0.01, and ^*∗∗∗*^*p* < 0.001 vs. the nontreated group).

Pharmacological network analysis identified critical targets for RR and HSCC, including CCNA2, TOP2A, AURKA, CCNB2, CDK2, and CHEK1. RT-PCR and Western blotting were utilized in this study to investigate the regulation mechanism of RR at the miRNA and protein levels, respectively. The results indicated that water extraction groups boosted TP53 expression while decreasing CCNA2, TOP2A, AURKA, CCNB2, CDK2, and CHEK1 expression (Figures 13 and 14).

## 4. Discussion

Esophageal squamous cell carcinoma is one of the most common histological subgroups of invasive solid tumors. Patients with advanced esophageal cancer often lose the opportunity of surgery and have a poor prognosis. Although there has been significant progress in immunotherapy for esophageal cancer, there is still a lack of effective drugs. At present, many traditional Chinese medicines have been proved to have inhibitory effects on esophageal squamous cell carcinoma [22, 23]. The composition of traditional Chinese medicine is complex, and the study of its mechanism is also complicated. Network pharmacology is a new methodology for investigating traditional Chinese medicine components and mechanisms [24–26]. In this study, the genes of ESCC were screened by bioinformatics, RR was screened by network pharmacology to target the hub genes of ESCC, and the inhibitory effect of RR on ESCC was studied *in vitro*.

WGCNA has been widely employed in biomedical research due to its excellent analytical performance. This is based on the hypothesis that molecules with comparable expression patterns may be involved in certain biological functions [27]. WGCNA quantifies the co-expression of molecules by calculating the expression correlation coefficient between them. Similar expression patterns in molecules may indicate that they are participating in the same biological activity or pathway. Thus, complicated omics data can be reduced to several functional modules, each of which can be connected with phenotypic information to identify physiologically meaningful modules. WGCNA has many advantages over an unweighted gene network [28]. To begin, it ensures the continued connectivity of network nodes. Second, it possesses exceptional analytical efficiency. In this paper, WGCNA was used to search for ESCC-related genes for subsequent network pharmacology analysis, aiming to find the action target of RR inhibiting ESCC more accurately.

In this article, network pharmacology analysis showed that RR's core targets for ESCC were CCNA2, CCNB2, CDK2, and CHEK1, and its inhibitory effect on ESCC was enriched in a variety of signaling pathways, mainly focusing on cell cycle signaling pathway and also affecting p53 signaling pathway. Our experiments demonstrated that RR water extracts arrested cell cycle and affected the expression of TP53, CCNA2, CCNB2, CDK2, and CHEK1 *in vitro* and were dose-dependent. Cell cycle arrest in response to DNA damage or cellular stress is crucial for genomic integrity to be maintained. Cell cycle checkpoints are critical in preventing cell cycle transition or triggering signal transduction in response to cell stress [29, 30]. The TP53 gene is one of the most significant tumor suppressor genes in humans, with cell cycle arrest being one of its primary regulatory activities [31]. P21 is the first transcriptional target of p53. P21 is capable of cell cycle arrest by inhibiting the production of cyclin/CDK complex [32]. Inactivation of the cyclin E/A (CCNA)-CDK2 and cyclin D-CDK4/6 complexes prevents the CDK-mediated phosphorylation of pRB, inhibiting the release of E2F transcription, which controls the transition from G1 to S phase [33, 34]. Thus, by increasing p53 expression, the p53 signaling pathway can be activated to induce cancer cell arrest in the G0/G1 phase, thereby limiting cancer cell proliferation. Reports from the real world have shown that the TP53 gene deletion rate correlates with esophageal cancer differentiation and lymph node metastasis [35]. TP53 gene mutation can lead to esophageal cancer progression [36–38]. Cyclins have long been thought to be important regulators of cell cycle progression. CCNA2 is a cyclin family member that is widely expressed [39]. It acts as an antagonist of P21 in cell cycle regulation [40]. Suppressing CCNA2 expression may enhance p53 expression, activating the p53 signaling pathway [41]. Immunopositivity to CCNA2 is related to advanced clinical staging and a low survival percentage in patients with ESCC [42]. The serine/threonine kinase CHK1, a key component of the DNA damage response, is encoded by CHEK1 [43]. CHK1 is also required for cell viability in the absence of DNA damage. CHK1 promotes the expression of p53-induced protein with death domain (PIDD) gene, resulting in caspase 2-mediated cell apoptosis [44, 45]. As a result, the authors hypothesized that RR inhibition of ESCC may affect the P53 signaling pathway via many targets, influencing the cancer cell cycle and decreasing cancer cell growth. Numerous articles have demonstrated that TOP2A and AURKA gene expression is increased in EC patients [46–49]. Topoisomerase II (TOP2A) is a gene encoding enzyme involved in DNA replication associated with anthracycline resistance in various cancers [50]. It is related to active mammalian cell proliferation and has been shown to have prognostic value in ESCC patients [47, 51]. AURKA is a member of the serine/threonine kinase family, which has been shown to act as an oncogene, promoting carcinogenesis in a variety of cancer types, including solid tumors and haematological malignancies [52]. A variety of AURKA kinase inhibitors (AKIs) have been developed during the last few decades to limit cancer cell proliferation, motility, and apoptotic invasion. P53 is a critical substrate for AURKA. Phosphorylation of AURKA can destabilize the expression of P53 protein and inhibit its anti-cancer activity [52]. Our *in vitro* experiments demonstrated that RR extracts reduced the expression of TOP2A and AURKA, suggesting the inhibitory effect of RR on ESCC.

WGCNA and network pharmacology were used in this study to investigate the anti-ESCC mechanism of RR and molecular docking technology, and *in vitro*, experimental verification proved that RR could inhibit the proliferation of ESCC by affecting the P53 signaling pathway and cell cycle pathway through multiple targets. These results shed fresh light on the clinical management of RR in ESCC. Additional in vivo tests can be performed to elucidate the mechanism by which RR inhibits ESCC.

## Figures and Tables

**Table 1:** The information of the bioactive compounds of *Rabdosia rubescens*.

No.	Molecule name	structure	PubChem ID	Molecular formula	Molecular weight (g/mol)	Degree value
1	Rubiadin	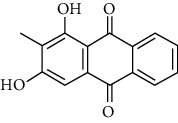	124062	C_15_H_10_O_4_	254.3	3
2	Sideritiflavone	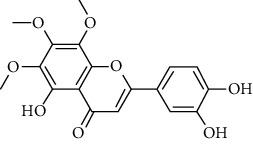	155493	C_18_H_16_O_8_	360.3	4
3	20-Hexadecanoylingenol	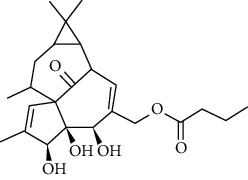	5318035	C_24_H_34_O_6_	418.5	5
4	Cinerins	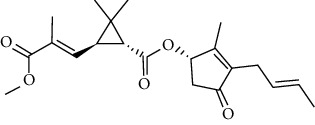	5281548	C_21_H_28_O_5_	360.4	6
5	Elemicin	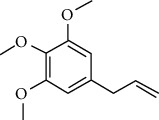	10248	C_12_H_16_O_3_	208.3	7
6	Dawoensin A	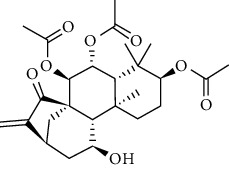	10412708	C_26_H_36_O_8_	476.6	3
7	Taipaienine	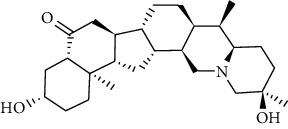	192522	C_27_H_43_NO_3_	429.6	4
8	Decanal	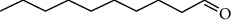	8175	C_10_H_20_O	156.3	2
9	Glabcensin V	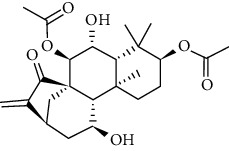	5317641	C_24_H_34_O_7_	434.5	4
10	Oleanolicacid	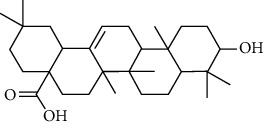	508-02-1	C_30_H_48_O_3_	456.7	3
11	Poricoic acid A	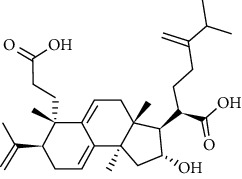	619166	C_31_H_46_O_5_	498.7	2
12	Taibairubescensin A	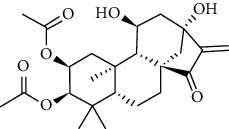	102237435	C_24_H_34_O_7_	434.5	8
13	Ursolicacidi	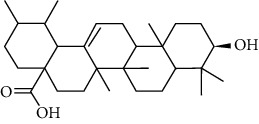	45358157	C_30_H_48_O_3_	456.7	2
14	Xindongnin A	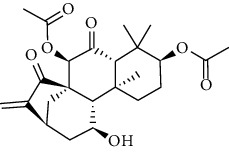	10410561	C_24_H_32_O_7_	432.5	6

**Table 2:** The bioactive compounds of molecular docking results.

Protein name	PDB ID	Test compounds	Binding energy (kcal/mol)
AURKA	MQ4	Rubiadin	−8.18
CCNA2	4EOP	Rubiadin	−7.86
CCNB2	5LQF	Sideritiflavone	−8.05
TTK	5N93	Rubiadin	−8.29
TOP2A	4R1F	Rubiadin	−8.56
CHEK1	2HY0	Rubiadin	−8.55
MELK	5TWL	Rubiadin	−8.02

## Data Availability

The data used to support the findings of this study are included within the article.
